# Structural basis for inhibition of the Tob-CNOT7 interaction by a fragment screening approach

**DOI:** 10.1007/s13238-015-0225-6

**Published:** 2015-10-30

**Authors:** Yuwei Bai, Shinya Tashiro, Satoru Nagatoishi, Toru Suzuki, Dongke Yan, Ruihua Liu, Kouhei Tsumoto, Mark Bartlam, Tadashi Yamamoto

**Affiliations:** State Key Laboratory of Medicinal Chemical Biology, Nankai University, Tianjin, 300071 China; College of Life Sciences, Nankai University, Tianjin, 300071 China; Medical Proteomics Laboratory, Institute of Medical Science, The University of Tokyo, 4-6-1 Shirokanedai, Minato-ku, Tokyo, 108–8639 Japan; Cell Signal Unit, Okinawa Institute of Science and Technology Graduate University, 1919-1 Onna-son, Kunigami, Okinawa 904-0412 Japan

**Dear Editor,**

The anti-proliferative protein Tob belongs to the Tob/BTG family (Matsuda et al., 2001) and plays important roles in cell proliferation, embryonic development, cellular differentiation, cancer suppression, and apoptosis (Matsuda et al., 2001; Iwanaga et al., 2003; Ito et al., 2005; Jia and Meng, 2007; Mauxion et al., 2009; Winkler, 2010). The anti-apoptotic and pro-survival effects of Tob may provide a tumor escape mechanism against chemo- and radiation therapies (Suzuki et al., 2012).

The anti-proliferative mechanism of Tob was revealed by its complex structure complex with CNOT7 (Caf1) (Horiuchi et al., 2009), and was further shown to be dependent on CNOT7 or CNOT8 but independent of other CCR4-NOT complex subunits (Doidge et al., 2012). Two conserved regions of Tob, termed Box A and Box B, mediate the largely hydrophobic interaction with CNOT7 and are conserved in other family members, including BTG2 (Yang et al., 2008). Several reports have suggested that Tob has no appreciable effect on the deadenylase activity of CNOT7 (Horiuchi et al., 2009; Ezzeddine et al., 2012), although the precise mechanism by which Tob regulates CNOT7 deadenylase activity remains unclear.

Protein-protein interactions play a crucial role in most biological processes and present attractive opportunities for therapeutic intervention (Pfaff et al., 2015). We employed a fragment screening approach to discover inhibitors of the Tob-CNOT7 interaction. Fragment screening is an alternative method to conventional high-throughput screening using small compounds of ~250 Da, which have many more desirable properties for the discovery of lead compounds than ~350 Da compounds used in conventional screening libraries. The low chemical complexity of fragments enables a small fragment library to cover more chemical space and yield a higher hit rate than conventional high-throughput screening libraries (Hann et al., 2001).

To identify chemical compounds that specifically bind to Tob, we screened 2000 fragments from The Drug Discovery Initiative (DDI) library that are soluble at 200 μmol/L in a buffer containing 5% DMSO. Tob stability was confirmed in a buffer containing 5% DMSO (Fig. S2). Initial screening was performed by surface plasmon resonance (SPR) on the CM5 sensor chip. The SPR response reflects the change of mass on chip surface directly, and is sensitive enough to detect binding of fragments to proteins on the chip (Giannetti et al., 2008). We selected specific binders based on the shape of the sensorgrams (Fig. 1A): sensorgrams with slow dissociation (signals are kept for 10 s after buffer injection) were treated as non-specific binding, while those with sample responses higher than 100 response units (RU) were treated as non-stoichiometric binding (Fig. 1B and 1C). After the removal of those binders, ~112 compounds exhibiting the top 5% response in each plate were selected as binding fragments to Tob (Fig. 1D and 1E).

**Figure 1:**
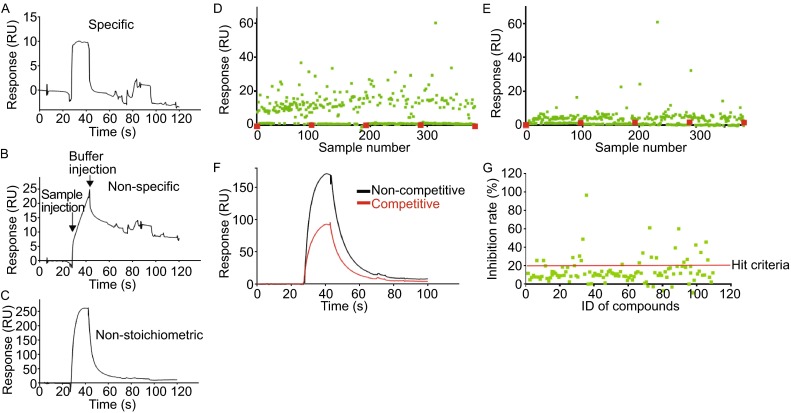
**Discovery of Tob-CNOT7 inhibitors by fragment screening**. (A–C) The binding analysis of compounds to Tob using Biacore shows three different responses. (A) Compounds showing responses of fast association and fast dissociation are treated as specific binding. (B) Compounds with responses of slow association and slow dissociation are treated as non-specific binding. The arrows indicate sample and buffer injection points as indicated. (C) Compounds showing responses higher than 100 RU are treated as non-stoichiometric binding. (D and E) Two representative results in the first round of screening for compounds that bind specifically to Tob. We tested one plate containing 384 compounds per day using SPR. Each green square corresponds to one compound and each red square corresponds to running buffer as a negative control. Non-stoichiometric bindings are not included. Compounds exhibiting the top 5% response in each plate were selected as binding fragments to Tob. (F and G) The screening for compounds that inhibit the Tob-CNOT7 interaction identified 20 compounds. (F) Competitive inhibition, as shown by a decrease in the response unit when the compound competes with CNOT7 for binding to Tob. (G) Compounds with  % inhibition higher than 20% (red line) are selected as hits

A second round of competitive screening was conducted to identify inhibitors of the interaction between Tob and CNOT7. Because the responses of the fragments were much smaller than the response of CNOT7 in SPR, the mixture of the inhibitor and CNOT7 shows a smaller response than CNOT7 alone. Each compound selected from the library of 2,000 compounds was mixed with CNOT7 and injected. Addition of some fragments resulted in a decrease of RU, suggesting that they inhibited the Tob-CNOT7 interaction (Fig. 1F). After the second round of screening, 20 compounds with an inhibition rate higher than 20% were selected as inhibitors of the interaction between Tob and CNOT7 (Fig. 1G). The structure of the hit compounds and their rates of inhibition are shown in Table S1.

To provide structural insight into fragment binding, crystals of human Tob residues 1–138 (termed TobN138 hereafter), containing Box A and B motifs, were soaked with a buffer containing several fragments. Structures of TobN138 with two inhibitors, corresponding to compounds 1 (i1) and 6 (i6) (Table S1), were determined to 2.3 Å resolution (Table S2). TobN138 consists of five α-helices and four β-strands that form two anti-parallel β-sheets. The highly conserved Box A region includes β1, α3, α2 and the connecting loop between them. The Box B region consists of the anti-parallel strands β2 and β3. The inhibitor-bound structures reveal two distinct binding sites in the CNOT7-binding interface of Tob (Fig. 2A and 2B).

**Figure 2:**
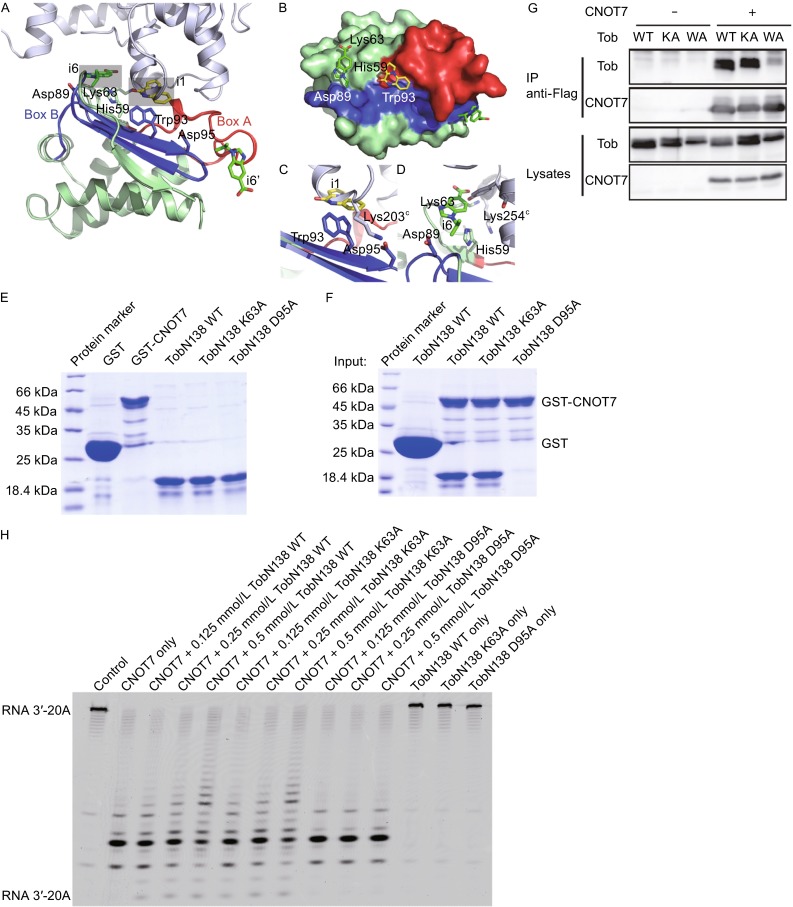
**Structural analysis of Tob inhibitors**. (A) Overall structure of Tob in complex with inhibitors 1 and 6. Tob is shown in pale green cartoon representation with the Box A and Box B motifs coloured red and blue, respectively. Inhibitors located in the CNOT7-binding interface of Tob are highlighted. The structure of the Tob-CNOT7 complex (PDB ID: 2D5R) is superimposed for comparison, with CNOT7 shown in pale blue cartoon representation. (B) Surface view of Tob shows the location of i1 and i6. The colour scheme is consistent with panel (A). (C) View of the i1 binding site showing the inhibitor overlapping with helix a10 of CNOT7. (D) View of the i6 binding site, showing the inhibitor overlapping with Tyr197^C^ of CNOT7 and Lys63 of Tob clashing with Lys254^C^ of CNOT7. (E and F) GST pull-downs using recombinant GST tagged CNOT7 and TobN138 (wild type, K63A, D95A mutants). GST served as a negative control. The migration of a molecular weight size marker is indicated. (G) Interaction of CAF1 with Tob wild-type and the mutants *in vivo*. Cos7 cells were transfected with Tob expression vector (WT, K63A, or W93A mutant) together with control or Flag-CNOT7 expression vector. Lysates were prepared from the cells and immunoprecipitated with anti-Flag antibody. The lysates and immunoprecipitates (IP) were analyzed by immunoblot using the indicated antibodies. (H) Deadenylase assay of CNOT7 with wild-type TobN138, K63A and D95A mutants *in vitro*. CNOT7 alone or CNOT7 with TobN138 wild-type, K63A and W95A mutant proteins were incubated with 5′-fluorescein isothiocyanate-labeled RNA substrate (RNA 3′-7 N + 20 As) for 30 min

In the TobN138-i1 complex, i1 π-stacks against Trp93 and is coordinated by the side-chain of Ser53. Superimposing the Tob-i1 complex onto the Tob-CNOT7 complex structure shows that i1 overlaps with Ser201 and Cys202 on helix α10 of CNOT7 (Fig. 2C). Trp93 is highly conserved among the Tob/BTG family and is located in the Box B motif (Fig. S1). Asp95 and Glu98 of Tob form a salt bridge with Lys203 in CNOT7, while Trp93 inserts into a hydrophobic pocket on the CNOT7 surface. A CNOT7 K203A mutant abrogates the interaction with Tob (Horiuchi et al., 2009), while BTG2 W103A and D105A mutants were unable to interact with CNOT7 (Yang et al., 2008). Trp103 and Asp105 of BTG2 are strictly conserved with residues Trp93 and Asp95 of Tob.

The i6 complex structure revealed two inhibitor molecules from the electron density map. The first is located in the CNOT7 interface close to Box A, where i6 is coordinated by Lys63 and Asp89 and stacks loosely against His59 (Figs. 2A and S3B). In the Tob-CNOT7 complex structure, Lys63 forms a number of contacts to the CNOT7 residues Tyr197 and Lys254, whereas residues His59 and Asp89 of Tob do not interact with CNOT7. Superposition of Tob-i6 and Tob-CNOT7 structures shows that i6 largely overlaps with Tyr197 in CNOT7. Lys63 of Tob adopts an extended side chain conformation in the inhibitor-bound structure and would clash with Lys254 in CNOT7 (Fig. 2D). His59 and Lys63 are located on the periphery of the Box A motif, with His59 located on strand β1 and Lys63 in the β1-α4 loop. Neither residue is strictly conserved in the Tob/BTG family (Fig. S1). Lack of sequence conservation in this site together with the low inhibition by i6 suggests it is dispensable for the CNOT7 interaction, but could be useful for design of inhibitors targeting specific Tob/BTG family members. The second inhibitor stacks against the aromatic side chain of Phe97, located on the β2-β3 loop in the Box B motif, and is further stabilized by a hydrogen bond between the O9 atom and the side chain of Glu47, in the Box A motif. Glu47 and Phe97 are strictly conserved in the Tob/BTG family, but this binding site is distant from the CNOT7 interface.

To test the importance of these two binding sites for interaction with CNOT7, two inhibitor binding residues (Lys63 in Box A, Trp93 in Box B) were individually mutated and the resulting mutant proteins were assayed for their ability to bind to CNOT7. Both the wild-type protein and K63A mutant were able to interact with CNOT7 *in vitro* (Fig. 2E and 2F), with respective dissociation constants (*K*_d_) of 3.25 × 10^7^ M^−1^ ± 2.18 × 10^7^ M^−1^ and 1.52 × 10^7^ M^−1^ ± 6.04 × 10^6^ M^−1^ as measured by isothermal calorimetry (ITC) (Fig. S4 and Table S3). Due to poor expression and stability of the Tob W93A mutant, we were unable to test the interaction of this mutant with CNOT7 *in vitro*, although the mutant expressed in mammalian cells for subsequent interaction assays *in vivo*. The equivalent BTG2 W103A mutant was also unstable, but a G64A-W103A double mutant could be expressed (Yang et al., 2008). However, expression of the equivalent Tob G54A-W93A double mutant also proved unsuccessful in this study. *In vivo* co-immunoprecipitation experiments of CNOT7 with wild-type Tob and the K63A and W93A confirmed that wild-type Tob and the K63A mutant, but not the W93A mutant, could interact with Flag-CNOT7 (Fig. 2G).

The effects of Tob or inhibitor binding on mRNA deadenylation mediated by CNOT7 were examined by *in vitro* deadenylase assays with single-strand poly(A) RNA. A D95A mutant was constructed as a negative control to substitute for W93A. The conserved Asp95 in Box B of Tob is equivalent to Asp105 of BTG2, which abrogates the interaction with CNOT7 (Yang et al., 2008). GST pull-down confirmed that Tob D95A was unable to interact with CNOT7 (Fig. 2E and 2F). When increasing amounts of wild-type and mutant Tob proteins were individually added to the CNOT7 deadenylase reaction system, the activity of CNOT7 in the presence of wild-type Tob and the K63A mutant was suppressed (Fig. 2H). No apparent differences were observed between CNOT7 alone and CNOT7 in the presence of the Tob D95A mutant (Fig. 2H).

Our study used a fragment-screening approach to identify a series of compounds that inhibit the Tob-CNOT7 interaction. Structural analysis of two fragment inhibitors delineated two distinct binding sites on Tob. The first shows 96.4% inhibition and binds to a strictly conserved site located on Box B in the CNOT7 interface, including residues Trp93 and Asp95. Missense mutations W93A and D95A abolish the interaction between Tob and CNOT7 *in vitro* and *in vivo*. The second inhibitor shows 43% inhibition and binds to a site close to Box A in the CNOT7 interface; mutation of Lys63 in this site reduces the binding affinity between Tob and CNOT7 to 47% of the wild-type. Our results indicate that Box B of Tob should be targeted for efficient inhibition of the Tob-CNOT7 interaction. Such inhibitors might prove useful as functional tools to probe the detailed biological role of Tob-CNOT7, or to specifically block the anti-apoptotic activity of Tob as a new avenue for development of anti-cancer therapeutics (Suzuki et al., 2012).

We further showed that Tob, but not the Tob D95A missense mutant, suppresses the deadenylase activity of CNOT7 *in vitro*. BTG2 also suppresses the activity of CNOT7 *in vitro*, while no appreciable effect on CNOT7 deadenylase activity was reported when a 4 molar excess of TobN138 was added (Yang et al., 2008; Horiuchi et al., 2009). In contrast, Tob1, Tob2, and BTG2 promote deadenylation and mRNA degradation in cellular transfection assays (Ezzeddine et al., 2007; Mauxion et al., 2009; Doidge et al., 2012; Ezzeddine et al., 2012). The underlying mechanism for this is unclear, but promotion by Tob of deadenylation and mRNA degradation *in vivo* may be linked to its interaction with PABP via its C-terminal PAM2 motifs (Ezzeddine et al., 2007), and Tob alone suppresses deadenylation and mRNA degradation. Further work is underway to test this hypothesis.

## Electronic supplementary material

Supplementary material 1 (PDF 1203 kb)
