# Supplementary material for: Structural basis for inhibition of the Tob-CNOT7 interaction by a fragment screening approach
Source: Protein Cell. 2015 Oct 30;6(12):924–8. doi: 10.1007/s13238-015-0225-6 (PMC4656213; doi:10.1007/s13238-015-0225-6)
Supplement: Supplementary file 1 — Supplementary material 1 (PDF 1203 kb) [file 13238_2015_225_MOESM1_ESM.pdf]

Structural basis for inhibition of the Tob-CNOT7 interaction by a fragment screening approach

Yuwei Bai[1,2*], Shinya Tashiro[3*], Satoru Nagatoishi[3], Toru Suzuki[4], Dongke Yan[1,2], Ruihua Liu[1,2], Kouhei Tsumoto[3†], Mark Bartlam[1,2†], & Tadashi Yamamoto[4]

Supplemental Material

Materials & Methods

Purification of TobN138 and CNOT7 for inhibitor screening

Human TobN138 cDNA was cloned into a modified pET-28b vector containing an N-terminal His6-tag and a SUMO cleavage site between the N-terminal tag and the inserted cDNA. *Escherichia coli* Rosetta2 (DE3) transformed with plasmid containing Tob was grown in 2xYT medium at 37°C. Isopropyl β-D-thiogalactopyranoside (IPTG) was added to the *E. coli* culture (1 mM) when OD600 reached to 0.6. Cells were grown for additional 24hr at 20°C and then harvested by centrifugation. The harvested cells were suspended in buffer A (20 mM Tris-HCl pH 7.9, 500 mM NaCl, 5 mM imidazole). Cells were homogenized with an ultrasonic cell-disruptor instrument (Tommy, Japan). The soluble fraction was separated by centrifugation and loaded into a nickel affinity chromatography (His-Trap HP, GE Healthcare). Protein was eluted with a gradient of 5-500 mM imidazole over 10 column volumes. The fractions containing TobN138 were pooled and subjected to a digestion with SUMO protease in buffer B (20 mM Tris-HCl pH 7.9, 150 mM NaCl) for 12h at 4 ºC. The digested products were loaded again in a nickel affinity chromatography to remove the cleaved His_6-tag. The fractions containing TobN138 were further purified on a size exclusion chromatography column (Hiload 16/600

Superdex 200 PG, GE Healthcare) equilibrated with buffer B. The fractions containing TobN138 monomer were used for the following assays.

Human CNOT7 cDNA was cloned into a modified pET-28b vector containing an N-terminal His6-tag. *E. coli* Rosetta2 (DE3) transformed with plasmid containing CNOT7 was grown in 2xYT medium at 37°C. IPTG was added to the *E. coli* culture (1 mM) when OD600 reached to 0.6. Cells were grown for additional 24hr at 20°C and then harvested by centrifugation. The harvested cells were suspended in buffer A. Cells were homogenized with an ultrasonic cell-disruptor instrument (Tommy, Japan). The soluble fraction was separated by centrifugation and loaded into a nickel affinity chromatography (His-Trap HP, GE Healthcare). Protein was eluted with a gradient of 5-500 mM imidazole over 10 column volumes. The fractions containing CNOT7 were pooled and further purified on a size exclusion chromatography column (Hiload 16/600 Superdex 200 PG, GE Healthcare) equilibrated with buffer B. The fractions containing CNOT7 monomer were used for the following assays.

Screening of fragments that bind to Tob

Fragment screening for Tob was conducted with a Biacore T200 instrument (GE Healthcare, Piscataway, NJ) using a CM5 sensor chip. First, Tob was immobilized on a CM5 sensor chip covalently by the amine coupling method in running buffer A (25 mM HEPES pH 7.5, 150 mM NaCl, 10 mM MgCl2 and 0.005% Tween 20). The immobilized level of Tob was about 7500 Response units (RU). The chemical compound library was supplied by the Drug Discovery Initiative (DDI), The University of Tokyo (http://www.ddi.u-tokyo.ac.jp/en/) and is

comprised of approximately 2000 fragment compounds. The compounds of this fragment library from DDI have the molecular weight of ~250 Da and the solubilities at 200 μM in the buffer containing 5% DMSO. The fragments were injected at 200 μM and a flow rate of 30 μl/min in running buffer B (25 mM HEPES pH 7.5, 150 mM NaCl, 10 mM MgCl2, 1 mM DTT, 0.005% Tween 20 and 5% DMSO). The binding level of each fragment was corrected with a standard solvent correction (4−6% DMSO in eight steps) in the Biacore T200 evaluation software.

Screening of compounds that bind competitively to Tob against CNOT7

Competitive screening for Tob was conducted with a Biacore T200 instrument (GE Healthcare, Piscataway, NJ) using a CM5 sensor chip. Tob was immobilized on a CM5 sensor chip covalently by amine coupling method in the running buffer A. Immobilized level of Tob was about 1000 RU. The fragments at 100 μM and CNOT7 at 0.3 μM were injected in the running buffer B and subsequently regeneration buffer (1 M Arg-HCl, pH 4.4) was injected at flow rate of 30 μl/min. As a positive control, CNOT7 at 0.3 μM was also injected in the first and final cycles. After solvent correction, the response of each samples was normalized to the positive control in the Biacore T200 evaluation software. The % inhibition is calculated by the following equation; the % inhibition =

[1 – (the response with compounds at 100 μM) / (the response without compounds)] x 100

Cloning, expression and purification for crystal screening and *in vitro* assays

To obtain the plasmids encoding GST-TobN138 and GST-CNOT7, the corresponding region of Tob and CNOT7 was separately PCR-amplified and cloned into the pGEX-6P-1 vector (Novagen) via BamHI and XhoI. The plasmids were then individually transformed into *E. coli* BL21 (DE3), which was expressed as a glutathione S-transferase fusion protein. Cells were cultured to an OD600 of 0.5 and induced with 0.5 mM IPTG for 18 h at 16 °C. After cell lysis, the GST-tagged TobN138 protein was applied to a glutathione affinity column (GE Healthcare) and eluted with PBS buffer. The glutathione S-transferase tag was then removed with PreScission Protease at 4 °C, and fractions containing the purest protein were pooled and concentrated (Amicon Ultra, Millipore) and buffer was exchanged with buffer A (20 mM Tris–HCl pH 8.0). For TobN138, protein was purified by anion exchange chromatography (Resource Q, GE Healthcare), eluting with a linear gradient of 0–1 M NaCl in buffer A. Fractions containing the purest recombinant TobN138 protein were pooled then subjected to gel filtration chromatography (Superdex-75 HR, GE Healthcare) using buffer B (20 mM Tris pH 8.0, 150 mM NaCl). The purity of resultant TobN138 was greater than ~95% as determined by SDS–PAGE analysis. To obtain the mutations of several key residues in the CNOT7-binding interface of human Tob, including K63A, W93A and D95A, single point mutations were introduced by the PCR method with the human TobN138 expression plasmid as a template using the Easy Mutagenesis System (TransGen) according to the manufacturer’s instructions. The TobN138 mutant proteins and CNOT7 were expressed and purified following the protocol for wild-type Tob described above.

Crystallization and data collection

The purified recombinant TobN138 protein was concentrated to 20 mg/ml in crystallization buffer (20 mM Tris–HCl pH 8.0). Optimal crystals were obtained using the hanging-drop, vapor-diffusion technique at 293K with reservoir solutions containing 0.1 M Citric acid pH 5.0, 3.0 M Sodium chloride and 3% v/v Ethylene glycol at a 1:1 ratio. To obtain inhibitor-bound complex structures, native TobN138 crystals were soaked at 293 K for 12 h in a solution containing inhibitor 1 or 6 at a final concentration of 1 mM in 0.1 M Citric acid pH 5.0, 3.0 M Sodium chloride and 5% DMSO. Crystals were flash-frozen in liquid nitrogen after soaking in a crystallization buffer containing 25 % glycerol as cryo-protectant. Data were collected at 100K on beamline BL17U of the Shanghai Synchrotron Radiation Facility. Data were processed with the HKL2000 suite of programs (Otwinowski and Minor, 1997).

Structure determination and refinement

Crystal structures of TobN138 were determined by molecular replacement using the previous crystal structure of Tob from the Tob-CNOT7 complex structure (PDB ID: 2ZI5) as a search model. The structures were refined by alternate cycles of manual rebuilding by Coot (Emsley et al., 2010) and refinement with PHENIX (Adams et al., 2010). Ligands were automatically fit into electron density using the LigandFit module in PHENIX and manually adjusted according to difference electron density maps during the late stages of refinement. Final structures were validated by MolProbity (Chen et al., 2010).

***In vitro* GST pull-down assays**

To obtain GST-tagged CNOT7, proteins were purified as mentioned above without removing the GST tag and exchanged with binding buffer (PBS supplemented with 5% glycerol). GST pull-down assays were performed in a final volume of 100 µl of the binding buffer using 5 µM of purified GST-tagged CNOT7 and TobN138 (wild type and mutants). The purified GST-tagged CNOT7 were incubated with glutathione beads for 30 min at 4 °C before adding TobN138 (wild type, K63A and D95A mutants) followed by another 2 hrs incubation at 4 °C. After washing five times with the binding buffer, the beads were analysed on a 13% SDS-PAGE gel.

ITC experiments

The binding affinities of TobN138 (wild type and K63A mutant) for CNOT7 were assayed with an ITC-200 microcalorimeter (MicroCal) device at room temperature. CNOT7 was placed in the reaction cell at a concentration of 0.05 mM in buffer C (20 mM HEPES pH 7.5, 150 mM NaCl with 5% DMSO). For each binding assay, TobN138 (wild type and K63A mutant) at a concentration of 0.45 mM were titrated into CNOT7 samples. The titration consisted of an initial injection of 0.4 µL followed by 20 injections of 1.5 µL every 150 s. The titration data and binding plot were analyzed with MicroCal Origin.

In vitro deadenylation assays

The purified and concentrated CNOT7 proteins (Figure 8a) were diluted in the deadenylase buffer and incubated with a poly(A) RNA substrate at 37 °C for 40 min. The wild type and mutant human Tob proteins and human CNOT7 were purified as described and dialyzed against the deadenylase buffer (50 mM Tris–HCl, 150 mM NaCl, 10% glycerol, 1 mM DTT, 5 mM $MgCl_2$ at pH 7.5). These proteins were incubated at 37 °C for 30 minutes with a synthesized poly(A) RNA substrate (5'-UCUAAAUAAAAAAAAAAAAAAAAAAA-3'; final concentration 0.5mM) labeled with fluorescein isothiocyanate at the 5' end (Morita et al., 2007). After addition of formamide, the reaction mixtures were fractionated on a 7 M urea-25% sequence polyacrylamide denaturing gel. The products were analyzed and quantified with a fluorescence imager, Typhoon Trio (GE).

References

Adams, P.D., Afonine, P.V., Bunkoczi, G., Chen, V.B., Davis, I.W., Echols, N., Headd, J.J., Hung, L.W., Kapral, G.J., Grosse-Kunstleve, R.W., *et al.* (2010). PHENIX: a comprehensive Python-based system for macromolecular structure solution. Acta Crystallogr D Biol Crystallogr 66, 213-221.

Chen, V.B., Arendall, W.B., 3rd, Headd, J.J., Keedy, D.A., Immormino, R.M., Kapral, G.J., Murray, L.W., Richardson, J.S., and Richardson, D.C. (2010). MolProbity: all-atom structure validation for macromolecular crystallography. Acta Crystallogr D Biol Crystallogr 66, 12-21.

Emsley, P., Lohkamp, B., Scott, W.G., and Cowtan, K. (2010). Features and development of Coot. Acta Crystallogr D Biol Crystallogr 66, 486-501.

Morita, M., Suzuki, T., Nakamura, T., Yokoyama, K., Miyasaka, T., and Yamamoto, T. (2007). Depletion of mammalian CCR4b deadenylase triggers elevation of the p27Kip1 mRNA level and impairs cell growth. In Mol Cell Biol, pp. 4980-4990.

Otwinowski, Z., and Minor, W. (1997). Processing of X-ray Diffraction Data Collected in Oscillation Mode. Methods in Enzymology 276, 307-326.

Supplementary Figures

Figure S1

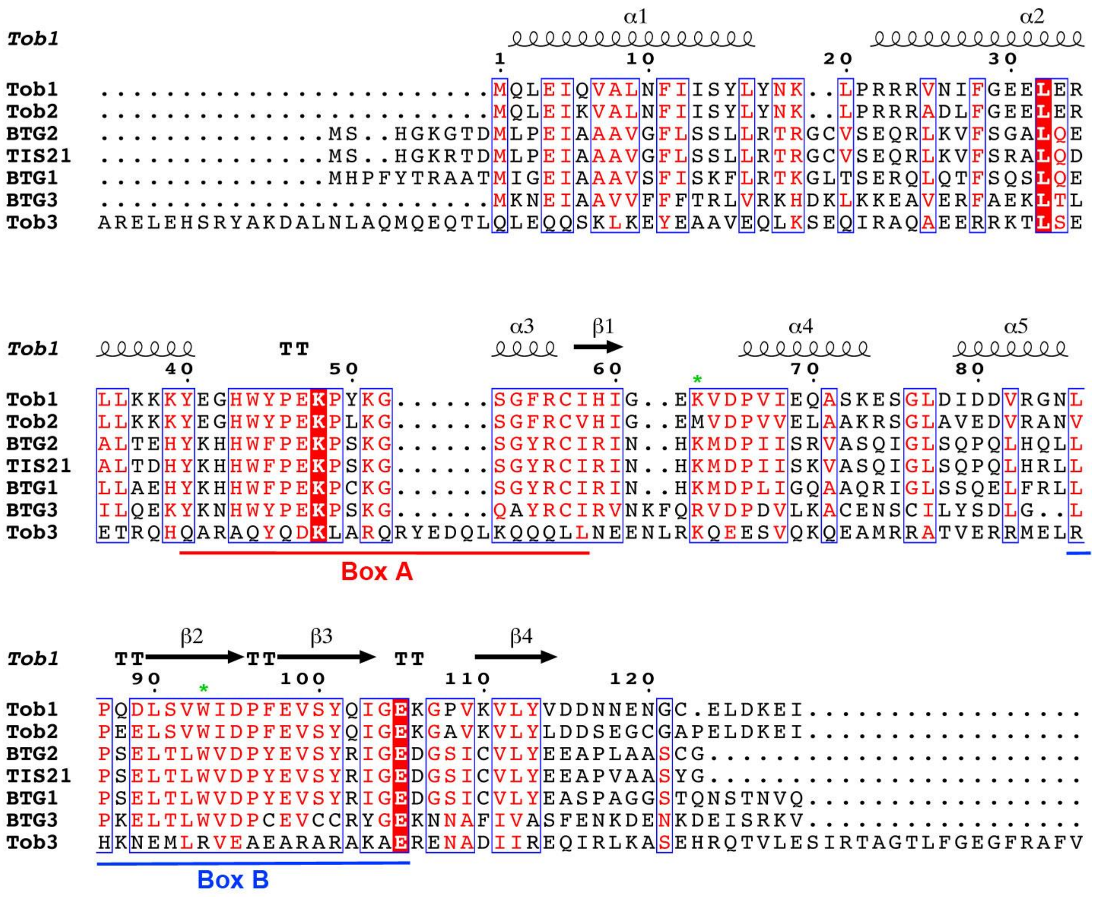

Multiple sequence alignment of *Homo sapiens* Tob1, Tob2, Tob3, BTG1, BTG2, BTG3 and *Mus musculus* TIS21 (sequences of Tob1, Tob2 and Tob3 are truncated for brevity). The Box A and Box B motifs are highlighted and residues Lys63 and Trp93 are indicated by a green asterisk. The sequence alignment was generated by ClustalW and displayed by ESPript.

Figure S2

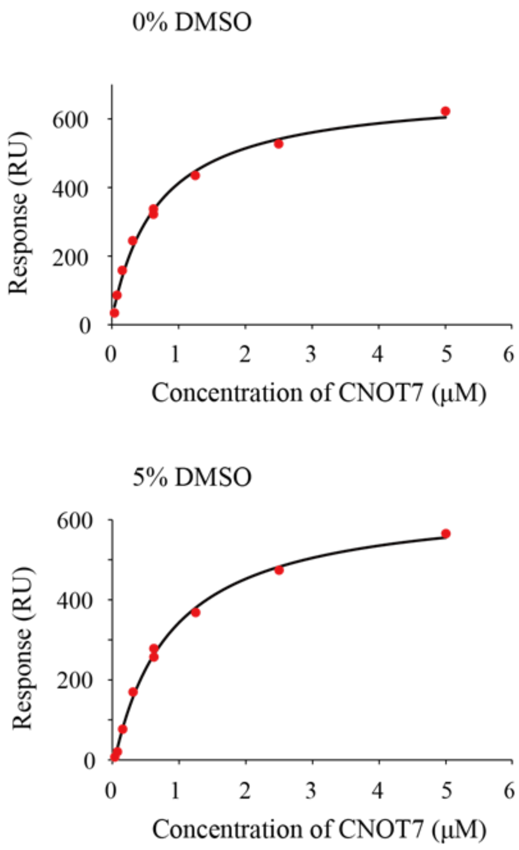

Tolerance of Tob stability in a buffer containing 0% DMSO (top) and 5% DMSO (bottom)

Figure S3

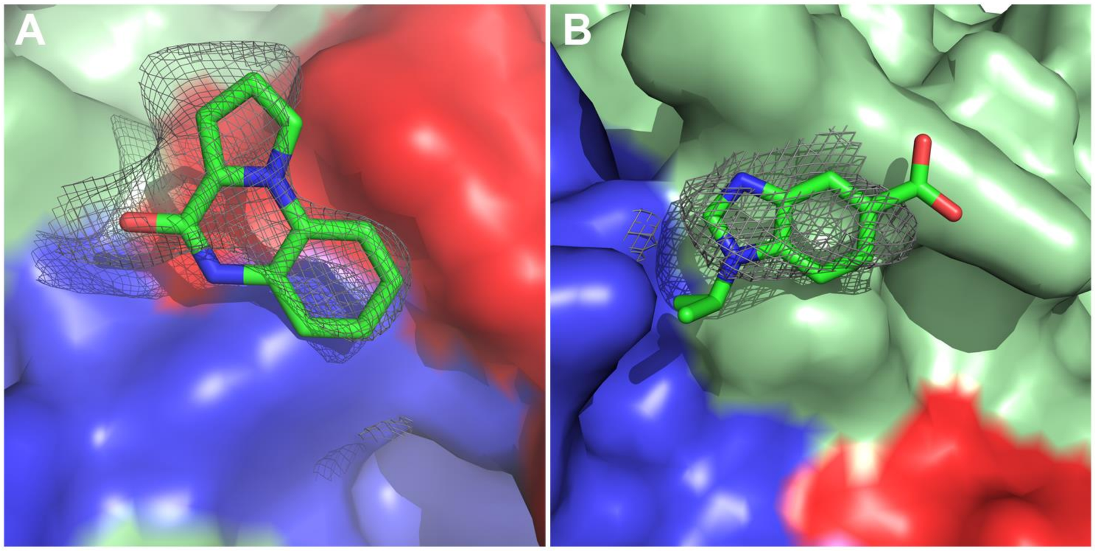

Electron density for (a) inhibitor 1 and (b) inhibitor 6. 2mFo-DFc composite omit maps are shown contoured at 1σ and coloured as a grey mesh. A surface representation of Tob is also shown and coloured according to the scheme in Figure 4 (Tob: pale green; Box A: red; Box B: blue).

Figure S4

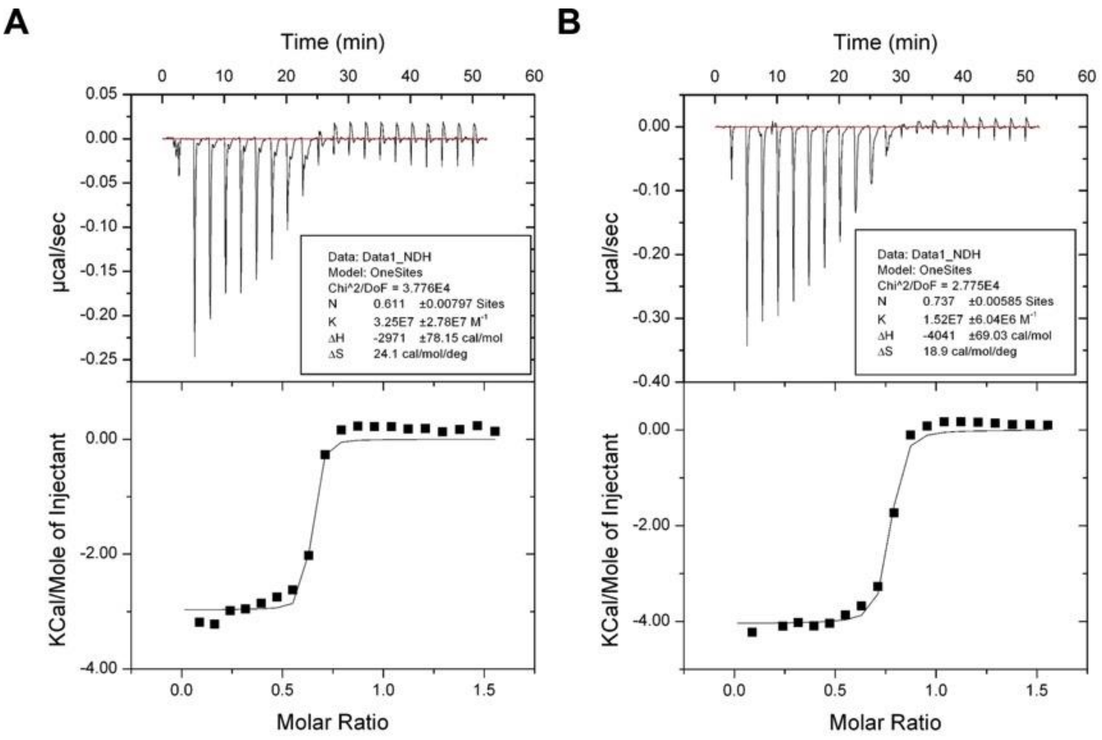

CNOT7 binding of TobN138 wild type and K63A mutant assayed with ITC. (a) CNOT7 titrated with TobN138 wild type. (b) CNOT7 titrated with TobN138 K63A mutant.

Figure S5

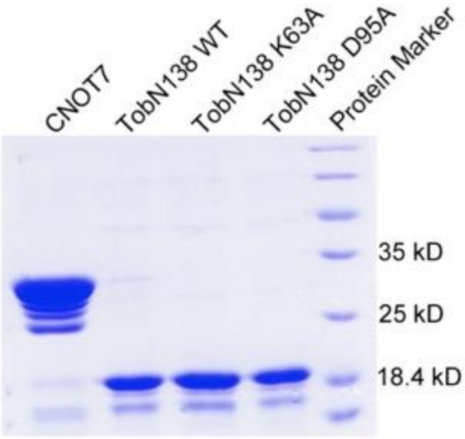

Protein profile of CNOT7, wild-type TobN138 and K63A, D95A mutants. The migration of a molecular weight size marker is indicated. According to the results of SDS-PAGE and UV spectrophotometer, proteins were diluted to the same concentration for deadenylase assay.

Table S1: List of hit compounds. The rate of inhibition of the Tob-CNOT7 interaction is shown for each compound at 100 μM.

Compound		% inhibition	Compound		% inhibition
1	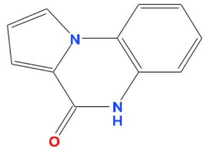	96.4	11	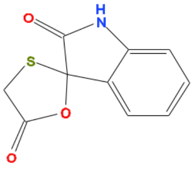	29.9
2	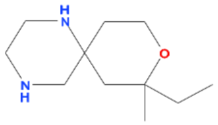	61.2	12	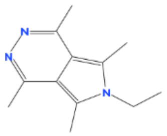	29.5
3	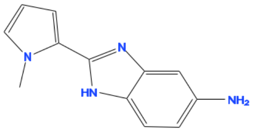	60.2	13	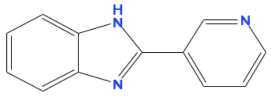	29.2
4	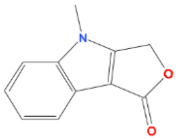	49.1	14	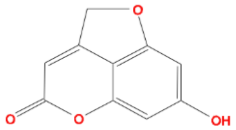	27.4
5	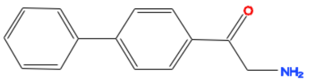	46.2	15	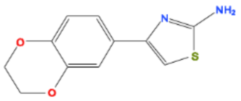	27
6	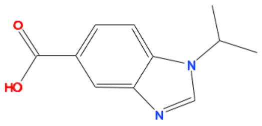	43.0	16	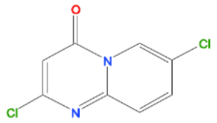	26.2
7	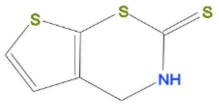	40.0	17	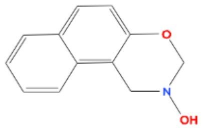	26.2
8	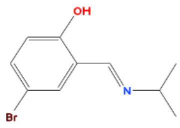	35.1	18	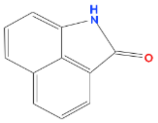	26.1
9	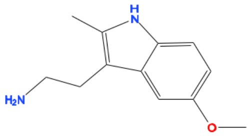	34.3	19	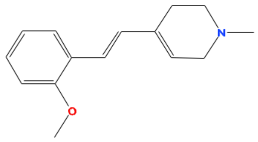	24.4
10	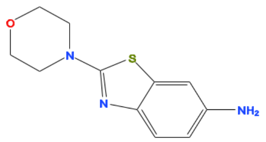	31.0	20	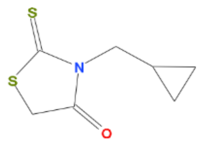	23.9

Table S2: Data collection and refinement statistics

Parameter	Tob-i1	Tob-i6
**Data collection**		
Space group	$P6_122$	$P6_122$
Cell dimensions a/b/c (Å)	65.0/65.0/163.7	65.1/65.1/162.9
Resolution (Å)	50-2.31 (2.35-2.31)	50-2.3 (2.35-2.30)
Average I/σ (I)[1]	52.6 (1.4)	32.6 (5.9)
Total reflections	110,471	180,073
Unique reflections	8,867	9,738
Completeness (%)[1]	94.4 (88.5)	99.6 (99.2)
Redundancy	12.5 (10.6)	18.5 (17.5)
R_{merge} (%)[1,2]	9.4 (59.6)	7.8 (48.9)
**Structure refinement**		
Resolution (Å)	50-2.3	50-2.3
R_{work}/R_{free} (%)[3]	23.2/27.8	21.4/26.5
R.m,s.d. bond lengths (Å)[4]	0.009	0.007
R.m.s.d. bond angles (°)[4]	1.479	1.020
Ramachandran plot		
Favored (%)	98.3	94.7
Outliers (%)	0	0
MolProbity score	2.19	2.20

[1] Numbers in parentheses correspond to the highest-resolution shell.

[2] $R_{merge} = \Sigma_i |I_i - \langle I \rangle| / \Sigma \langle I \rangle$, where I_i is an individual intensity measurement and $\langle I \rangle$ is the average intensity for all the reflections.

[3] $R_{work}/R_{free} = \Sigma ||F_o|-|F_c|| / \Sigma|F_o|$, where F_o and F_c are the observed and calculated structure factors, respectively.

[4] R.m.s. deviations relate to the Engh and Huber parameters.

Table S3: Interaction properties between Tob and CNOT7 calculated by ITC.

**Tob**	**K_d (M^{-1})**	**N (sites)**
Wild-type	3.25E7 ±2.18E7	0.611 ±0.00797
K63A	1.52E7 ±6.04E6	0.737 ±0.00585
